# Management of Impacted Primary Molar Obstructing Permanent Tooth Eruption in a Pediatric Patient: A Case Report

**DOI:** 10.7759/cureus.75480

**Published:** 2024-12-10

**Authors:** Anuj Jain, Saumya Taneja, Vaishnavi Singh, Ankita Chandak

**Affiliations:** 1 Oral and Maxillofacial Surgery, Geetanjali Dental and Research Institute, Udaipur, IND; 2 Pediatric and Preventive Dentistry, Inderprastha Dental College, Ghaziabad, IND; 3 Pediatric and Preventive Dentistry, Institute of Dental Sciences, Bareilly, IND; 4 Pediatric and Preventive Dentistry, Dr. Hedgewar Smruti Rugna Sewa Mandal's Dental College and Hospital, Hingoli, IND

**Keywords:** delayed eruption, impacted primary tooth, impacted tooth, primary molar, surgical extraction

## Abstract

Tooth impaction and eruption failure present common challenges in pediatric dentistry. We report a case of a 10-year-old boy of Indian origin presenting with a missing left mandibular primary second molar and impacted first permanent molar. Radiographic examination revealed an ankylosed primary molar obstructing the path of an unerupted premolar. Surgical extraction under local anesthesia was performed successfully, with postoperative management, including antibiotics and analgesics. Follow-up examinations demonstrated favorable outcomes, prompting referral to an orthodontist for further alignment. This case underscores the importance of timely intervention and interdisciplinary collaboration in managing dental impactions to ensure optimal dental health and development in pediatric patients.

## Introduction

Tooth impaction is a condition in which the tooth fails to erupt from its site of formation to its functional position in the alveolar process beyond the normal time of the eruption. Impaction can be categorized as either primary, where the tooth has not erupted ever, or secondary, where the tooth gets impacted again due to one of the numerous factors. Primary impaction is usually asymptomatic and is mostly diagnosed accidentally on radiographs. Secondary impaction can be an outcome of local or systemic factors such as ankylosis, odontomas, trauma, inadequate space, over-retained primary teeth, dilacerations, heredity, postnatal tuberculosis, anemia, malnutrition, endocrinal disorders, and developmental anomalies such as cleft palate [1,2].

The most commonly impacted permanent teeth are third molars followed by mandibular second premolars. Impaction of primary teeth is a rare entity, with a reported prevalence rate of 1.3%-8.9% of the population [3,4]. Among primary teeth, mandibular and second molars are most frequently impacted [5].

Unerupted primary teeth may cause complications such as space loss and tipping of the adjacent teeth, resulting in impaction or change in the path of eruption of permanent teeth or the supra-eruption of the antagonist tooth [2,6]. Therefore, to avoid the ramifications, management of impacted primary tooth is a must. Treatment options available are follow-up without treatment, surgical exposure with or without orthodontic traction, or complete surgical removal of the tooth [7]. We hereby present a case of a 10-year-old child with a submerged mandibular primary second molar and its management.

## Case presentation

**Figure 1:**
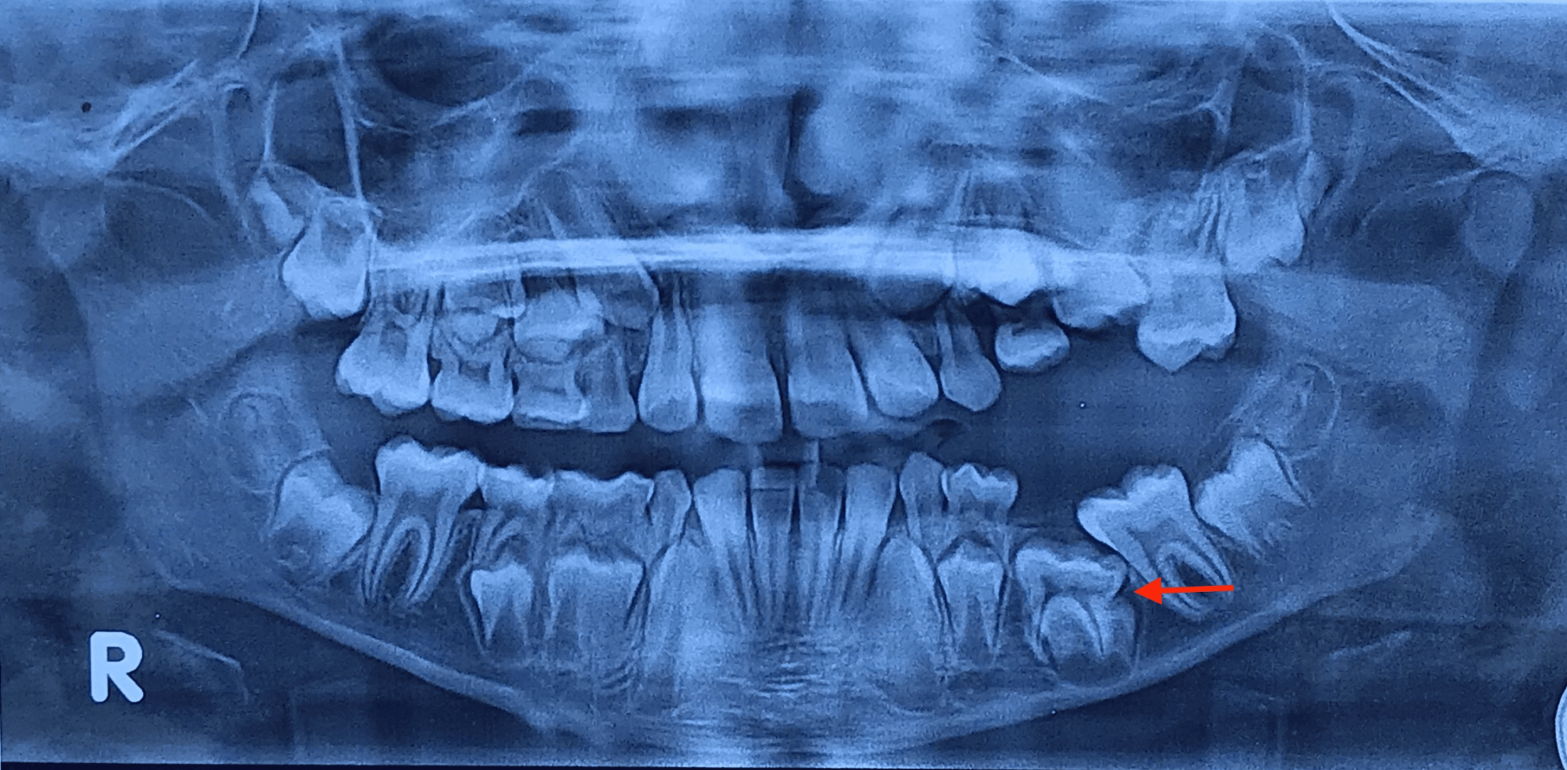
Pre-operative orthopantomogram showing impacted mandibular primary left second molar

A 10-year-old boy of Indian origin presented at a private dental clinic with the chief complaint of a missing tooth on the left side of his lower jaw. He reported no pain, discomfort, or other associated symptoms. Clinical examination revealed the absence of the left mandibular primary second molar and the permanent first molar on the same side. Radiographic examination, specifically an orthopantomogram (Figure 1), revealed an impacted mandibular second primary molar obstructing the path of the developing tooth bud of the second premolar and the first permanent molar.

**Figure 2:**
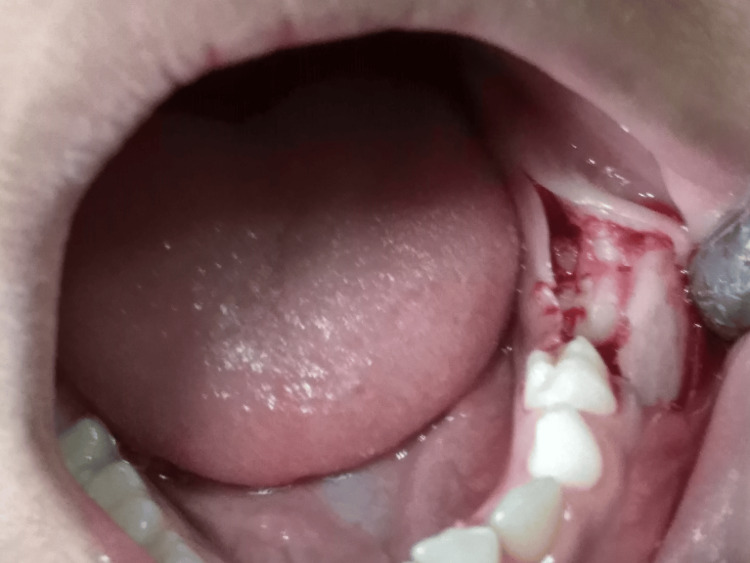
Intra-operative photograph showing incision, flap reflection, and bone guttering to expose the tooth

**Figure 3:**
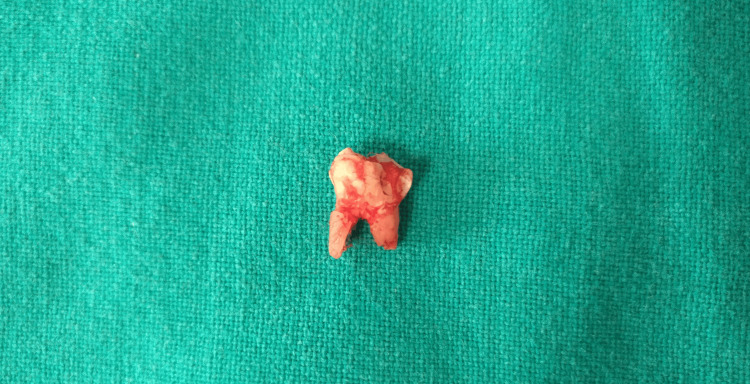
Extracted primary second molar

**Figure 4:**
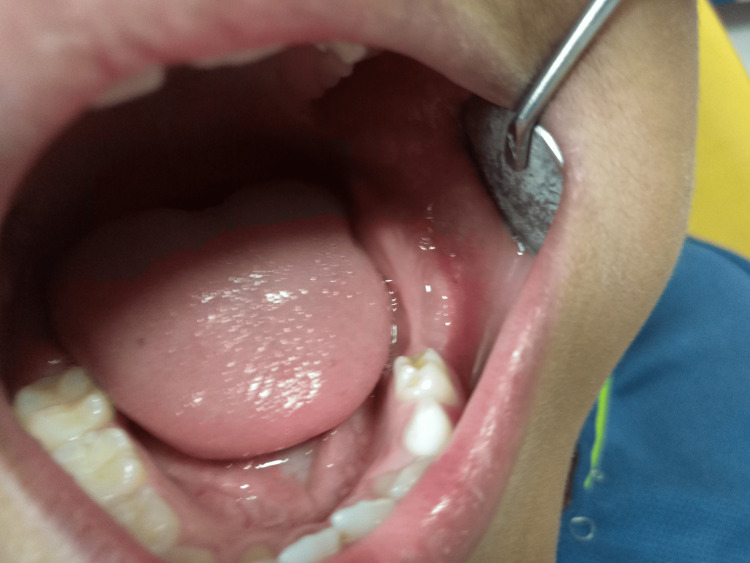
Post-operative photograph showing satisfactory healing and no post-operative complications

Based on the radiographic findings, it was decided to surgically extract the impacted primary tooth. After obtaining consent from the parents, the procedure was performed under aseptic conditions. Local anesthesia was administered, a crestal incision was made along the left mandibular ridge, and a full-thickness mucoperiosteal flap was elevated (Figure 2). Using a round Ni-Ti bur, the overlying bone was carefully removed to expose and subsequently extract the impacted primary tooth (Figure 3). The flap was repositioned and closed using 3-0 black silk sutures. Postoperatively, the patient was prescribed amoxicillin 250 mg twice daily and ibuprofen 200 mg twice daily for 5 days. He was recalled after a week for suture removal, during which no postoperative complications were noted (Figure 4).

**Figure 5:**
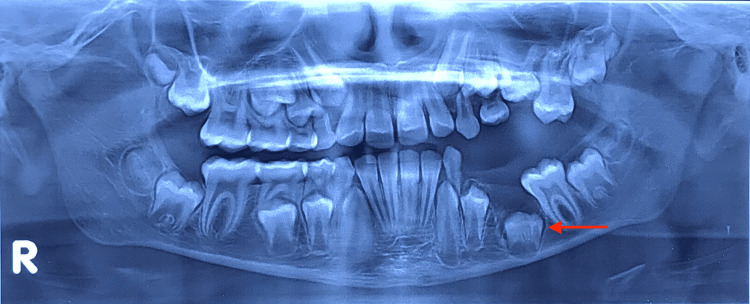
Three months’ post-operative orthopantomogram showing underlying premolar following the path of eruption

**Figure 6:**
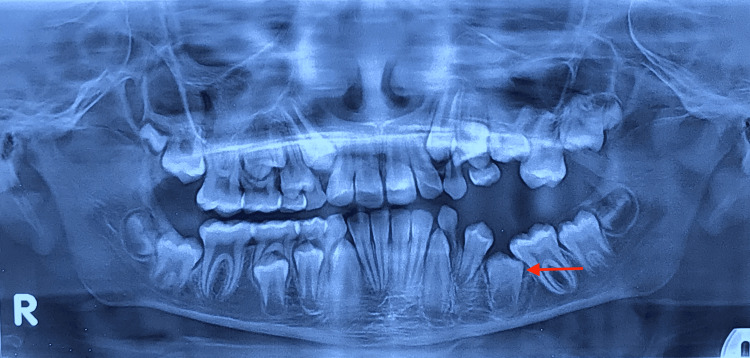
Nine months’ post-operative orthopantomogram showing underlying premolar following the path of eruption

The patient was kept under observation and underwent periodic radiographic and clinical examinations. The first permanent molar was in Nolla's stage 9. Considering the age of the patient, we waited for the tooth to erupt in the oral cavity and planned to use the long band and loop space maintainer. After 3 months, a follow-up radiograph (Figure 5) revealed that the second premolar was progressing along its normal path of eruption, and the first primary molar was exfoliated. After 9 months, another orthopantomogram was taken (Figure 6), which showed continuous root formation of the second premolar. The first molar had yet to erupt into the oral cavity. Consequently, the patient was referred to an orthodontist for further management and proper alignment of teeth within the arch. The patient was lost to follow up after this.

## Discussion

Problems related to tooth impaction and failure of eruption are commonly encountered in dental practice. Literature indicates that impaction affects males and females equally, with no significant difference in prevalence between the right and left sides [1]. Impaction in primary dentition is rare, accounting for 2.5% to 8.3%, and cases where impacted primary molars are positioned inferior to unerupted premolars are even less frequent [8].

In some instances, early ankylosis of a primary molar can cause subsequent premolars to assume a superior and lateral position relative to their primary predecessor, as reported by Borsatto et al. [8]. However, in our case, the impacted primary tooth was positioned superior to the underlying unerupted premolar. Over time, ankylosed unerupted teeth become increasingly embedded due to bone deposition, leading to potentially serious complications and necessitating prompt treatment.

Treatment considerations depend on several factors, including the characteristics of the impacted tooth, such as angulation, position, root structure, presence or absence of root pathologies, available space, presence of associated pathologies, and its relation to developing permanent tooth buds. When there is sufficient space for eruption, normal axial inclination, and no morphological abnormalities, conservative surgical exposure of the tooth is recommended, involving excision of overlying soft tissues, bone, or any other pathology [1]. The impacted tooth is then monitored, and if spontaneous eruption does not occur within 3 months, orthodontic traction may be initiated. In cases where the eruption is unlikely due to ankylosis or abnormal eruption path or where there is a risk of cysts or tumors developing, extraction of the tooth and provision of a space maintainer is recommended [1]. Sakai et al. suggested fenestration of overlying bone and tissue prior to initiating traction [9]. Some researchers advocate prolonged observation for spontaneous eruption of premolars, as delay in their eruption is common and may continue beyond 10 years of age due to delayed mineralization of unerupted primary predecessors [7].

Coronectomy is another treatment option considered in cases where impacted primary molars are close to the underlying nerve or pose a risk of mandibular fracture [10]. However, this option was not applicable in our case, as no such complications were observed.

In our case, radical surgical treatment was chosen as the preferred approach because the spontaneous eruption was deemed improbable, and the impacted tooth was obstructing the path of eruption for permanent teeth. To prevent potential pathological complications, a complete extraction of the tooth was successfully performed. The patient has been referred to an orthodontist to facilitate orthodontic extrusion of the first permanent molar, given the minimal likelihood of spontaneous eruption.

## Conclusions

In conclusion, timely surgical extraction of the impacted primary molar effectively resolved obstruction to the eruption path of permanent teeth in our pediatric patient. Postoperative recovery was uneventful, supported by antibiotics and analgesics, with subsequent radiographic assessments confirming successful treatment outcomes. Referral to orthodontic care ensures continued monitoring and alignment of dentition. This case emphasizes the critical role of early intervention and interdisciplinary collaboration in achieving optimal dental health outcomes in pediatric dentistry.
